# Chronic Exposure to Dim Light at Night or Irregular Lighting Conditions Impact Circadian Behavior, Motor Coordination, and Neuronal Morphology

**DOI:** 10.3389/fnins.2022.855154

**Published:** 2022-04-13

**Authors:** Tara C. Delorme, Shashank B. Srikanta, Angus S. Fisk, Marie-Ève Cloutier, Miho Sato, Carina A. Pothecary, Chantal Merz, Russell G. Foster, Steven A. Brown, Stuart N. Peirson, Nicolas Cermakian, Gareth T. Banks

**Affiliations:** ^1^Department of Psychiatry, Douglas Mental Health University Institute, McGill University, Montréal, QC, Canada; ^2^Nuffield Department of Clinical Neurosciences, Sleep and Circadian Neuroscience Institute, University of Oxford, Oxford, United Kingdom; ^3^Chronobiology and Sleep Research Group, Institute of Pharmacology and Toxicology, University of Zurich, Zurich, Switzerland; ^4^Mammalian Genetics Unit, MRC Harwell Institute, Oxfordshire, United Kingdom

**Keywords:** circadian disruption, light at night, aging, behavior, bioluminescence, dendritic morphology

## Abstract

Mistimed exposure to light has been demonstrated to negatively affect multiple aspects of physiology and behavior. Here we analyzed the effects of chronic exposure to abnormal lighting conditions in mice. We exposed mice for 1 year to either: a standard light/dark cycle, a “light-pollution” condition in which low levels of light were present in the dark phase of the circadian cycle (dim light at night, DLAN), or altered light cycles in which the length of the weekday and weekend light phase differed by 6 h (“social jetlag”). Mice exhibited several circadian activity phenotypes, as well as changes in motor function, associated particularly with the DLAN condition. Our data suggest that these phenotypes might be due to changes outside the core clock. Dendritic spine changes in other brain regions raise the possibility that these phenotypes are mediated by changes in neuronal coordination outside of the clock. Given the prevalence of artificial light exposure in the modern world, further work is required to establish whether these negative effects are observed in humans as well.

## Introduction

Circadian clocks are internal timing systems that allow organisms to adjust their behavioral and physiological rhythms to anticipate daily changes in the environment. Circadian clocks generate self-sustained oscillations at the cellular, tissue and behavioral levels, and are present in most tissues and cell types (Dibner et al., 2010). The rhythm-generating mechanism is based on a gene expression network with a delayed negative feedback loop that causes levels of affected transcripts and proteins (estimated to be 5–20% of all such molecules in any tissues) to oscillate with a period of approximately 24 h (Takahashi, 2017). In mammals, the master regulator of these processes is the suprachiasmatic nucleus (SCN), a small bilateral nucleus present in the anterior hypothalamic region of the brain (Dibner et al., 2010). Although self-sustained, all circadian clock-generated rhythms, including those of the SCN, can be entrained by rhythmic environmental cues (Hastings et al., 2019). For the SCN clock, the strongest entraining cue (“*Zeitgeber”*) is the light-dark cycle (Czeisler et al., 1986; Duffy and Wright, 2005; Lucas et al., 2014).

In the retina, a specific subset of ganglion cells known as intrinsically photosensitive retinal ganglion cells (ipRGCs) express the blue-light sensitive photopigment melanopsin. These cells provide the primary pathway by which light information is transmitted to the SCN (Berson et al., 2002; Hattar et al., 2002; Guler et al., 2008). Not only do the ipRGCs innervate the SCN, but they also innervate other regions of the brain such as the olivary preoptic nucleus (OPN), which mediates pupil constriction, in an intensity-dependent manner (Lucas et al., 2001). The retinohypothalamic tract, which provides light input from the retina, innervates the ventrolateral region of the SCN (Ebling, 1996). Light input from the retina involves several signaling pathways and lead to the induction of many genes, including *c-fos* and clock genes *Per1* and *Per2*, in this region of the SCN (Meijer and Schwartz, 2003). These molecular events entrain the central clock machinery to the light input from the external environment (Ikonomov et al., 1994; Golombek and Rosenstein, 2010).

The SCN drives rhythms in behavior and physiology through the modulation of various physiological processes such as body temperature, hormones, and metabolism, and by synchronizing oscillators in peripheral organs (Dibner et al., 2010). It is thus unsurprising that disruption of these rhythms has direct consequences on human health parameters (such as cardiovascular health and body weight), and is associated with a higher risk of developing mental health disorders (including mood disorders) and physiological diseases (such as diabetes and hypertension) (Evans and Davidson, 2013). This is especially concerning in the modern day when light exposure is no longer limited to the sun-imposed “daytime”. In our 24-h society, with the prevalence of electrical lighting and fast-paced advancement of technology, light exposure at inappropriate times of day is increasingly common. Such mistimed lighting events can include chronic night-time light exposure (by electrical lighting and electronic devices before bed) or chronic irregular exposure to light-dark cycles (for example the differences in sleep and wake times during the work week compared to the weekend). The detrimental effects of mistimed light exposure have been well documented in a plethora of studies conducted on different sub-sections of the population (Evans and Davidson, 2013; Cho et al., 2018; Blume et al., 2019). Comparative work in animal models has further demonstrated such detrimental effects (Castanon-Cervantes et al., 2010; McGowan and Coogan, 2013; Deibel et al., 2014; Inokawa et al., 2020) and older animals show increased severity of these effects (Davidson et al., 2006). However, such studies have generally been performed using abnormal light exposures of 12 weeks at most. While some investigations of long-term disruptive lighting have been performed, very disruptive conditions to which a normal mouse cannot entrain its clock were used (Spoelstra et al., 2016; Inokawa et al., 2020). Comparatively little is known about long-term exposure to more subtle disruptive conditions such as dim light at night or social jetlag, which may be more representative of the impact of the modern living conditions on most of the population.

Human and rodent studies have shown that aging affects the circadian system (Buijink and Michel, 2021). While there is an age-related reduction in light sensitivity (Lupi et al., 2012), melanopsin-mediated responses appear to be retained in older animals even in the absence of rods and cones (Semo et al., 2003). Age-related changes have been widely reported in circadian rhythms and sleep, although there are conflicting reports as to the phenotypic effects of aging on the central clock itself (Buijink and Michel, 2021). Interestingly, the circadian clock itself also affects aging: transplanting the SCN of young mice into old mice resulted not only in increased robustness of circadian rhythms (Van Reeth et al., 1994), but also in an increase in life expectancy (Hurd and Ralph, 1998). It is still unknown what the relationship between aging and long-term abnormal lighting is, and whether these detrimental effects can be prevented or reversed.

The aim of this study was to analyze the effects of long-term exposure to abnormal lighting conditions in mice. We explored retinal sensitivity to light and light input to the SCN using pupillometry and cFos expression, respectively. We also explored the functioning of the SCN clock itself by analyzing endogenous rhythms of the SCN using PER2::LUC reporter mice. Additionally, we explored circadian output by characterizing behavioral rhythms in wheel running and passive infrared sensor-based activity. Finally, we conducted a measure of neuronal morphology (quantification of dendritic spines), behavior (including tests of motor function, anxiety-like behavior, cognition, and sensory motor gating) and physiology (clinical chemistry analysis). Our data indicate sustained effects of chronic abnormal lighting, especially dim light at night (DLAN), on behavior and brain function.

## Materials and Methods

### Mice

Male C57BL/6J mice (Jackson Laboratories, product number: 000664) and male mice that were heterozygous for the PER2::LUC allele (Jackson Laboratories, product number: 006852) on a C57BL/6J background were used. All mice were weaned at 3 weeks of age, and group housed (no more than 5 mice per cage) until adulthood (8 weeks) in a standard 12 h:12 h light:dark cycle (with cool white LED lighting at ∼200 lux). Starting at ∼10 weeks old, mice were subjected to chronic disruption by abnormal lighting conditions. After the chronic light disruption, mice used for wheel running analysis and passive infrared recordings were singly housed, while all other measurements allowed for continuous group housing. Mice used for non-circadian behaviors were either C57BL/6J mice (grip strength, elevated plus maze, Morris water maze, prepulse inhibition of acoustic startle) or PER2::LUC mice (rotarod, open field). For euthanasia, the bioluminescence experiments required cervical dislocation without anesthesia, while all other experiments allowed for cervical dislocation with anesthesia by isoflurane inhalation, followed by CO_2_ euthanasia. All efforts were made to minimize suffering.

Mouse cohorts used for non-circadian behaviors, wheel running activity and *ex-vivo* bioluminescence experiments were housed at the Douglas Mental Health University Institute, Montreal, QC, Canada. These procedures were carried out in accordance with guidelines of the Canadian Council on Animal Care and approved by the Animal Care Committees of McGill University and Douglas Mental Health University Institute. Mouse cohorts used for passive infrared recordings (PIR), light-induced cFos expression, pupillometry, body temperature recordings, clinical chemistry and dendritic spine analysis were housed at the University of Oxford and performed under the UK Animals (Scientific Procedures) Act 1986 under PPL PE4ED9D2C in accordance with the University Policy on the Use of Animals in Scientific Research.

In both facilities, the mice were maintained in standard group-housing cages with *ad libitum* access to normal chow and water. All animals received light from a cool white LED lighting source, with all the light measurements carried out at the level of the animals (base of cage).

### Chronic Disruption by Abnormal Lighting Conditions

**FIGURE 1:**
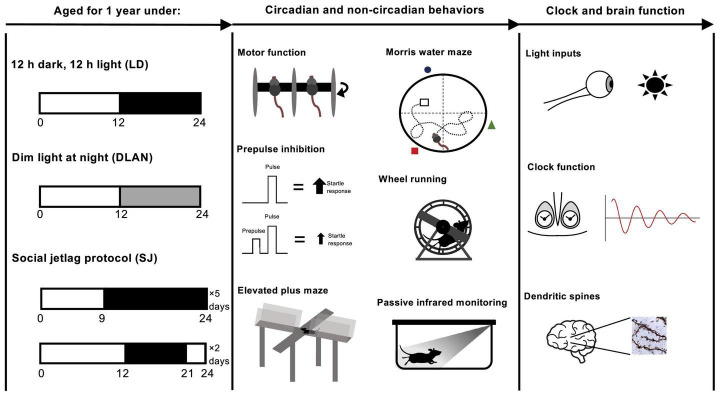
Experimental protocol for the aging of mice over the span of 1 year, under different lighting conditions followed by mouse phenotyping. Starting at ∼8 weeks of age, mice were left to habituate to the light-proof cabinets. Then, at ∼10 weeks old, mice were subjected to one of the following three abnormal lighting condition for 12 months (i.e., until ∼62 weeks old): (1) standard 12:12 light-dark cycles (light at ∼200 lux) (LD group, control); (2) 12:12 light cycles in which the dark phase is replaced by one of dim light (light phase light at ∼200 lux, dim light at ∼20 lux) (dim light at night (DLAN) group); (3) an environment of 5 days 9:15 L:D and 2 days 15:9 L:D per week (social jetlag (SJ) group). Following the 12-month chronic disruption period, mice were returned to standard 12:12 LD cycles for at least 2 weeks prior to testing.

At ∼8 weeks old, mice were transferred into light-proof cabinets [either custom build (Oxford) or from Actimetrics, IL, United States (McGill)] and subjected to 12:12 LD for a 2-week habituation phase, before they underwent 12 months of abnormal lighting exposure. Mice were subjected to one of the following three lighting conditions for a period of 12 months: (1) standard 12 h:12 h light-dark cycles (light at ∼200 lux) (LD group); (2) 12 h:12 h light cycles in which the dark phase is replaced by one of dim light (light phase light at ∼200 lux, dim light at ∼20 lux) (dim light at night (DLAN) group); (3) an environment of 5 days 9 h:15 h L:D and 2 days 15 h:9 h L:D per week (social jetlag (SJ) group) (Figure 1). Following the 12-month chronic disruption period, mice were returned to standard 12:12 LD cycles for a period of at least 2 weeks prior to phenotyping.

### Wheel Running Activity

After the 2-week acclimatization period in LD, post-light disruption, wheel running activity analysis was performed as previously described (Brown et al., 2019; Delorme et al., 2021). Briefly, mice were individually housed in running wheel cages, which were placed in light-proof ventilated cabinets. Light (∼200 lux) was controlled via an external timer. After a 2-week acclimatization and entrainment period, mice were exposed to 3 lighting conditions, each for 3 weeks: 12:12 LD, constant darkness (DD) and constant light (LL) (*n* = 7–12 per group). Running wheel activity data was collected and analyzed using ClockLab software, version 6 (Actimetrics, Wilmette, IL, United States). Analysis was carried out on the last 10 days of wheel-running recordings for each condition. We calculated various circadian locomotor activity variables, including circadian period (tau; calculated using a chi-square periodogram), duration of the active period (*alpha*; denoted by the numbers of hours between activity onset and offset), total amount of daily activity and percent day activity relative to total daily activity. In constant conditions (DD and LL), the subjective night was defined to be from the beginning of activity onset until half a period later, and the rest of the circadian cycle was defined as the subjective day. Analyses for non-parametric variables included interdaily stability, which quantifies the synchronization to the 24 h light–dark cycle, intradaily variability, which quantifies rhythm fragmentation, and relative amplitude, which quantifies the robustness of the rhythm (Van Someren et al., 1999). A detailed analysis of activity bouts was also done, where a bout is defined as a sustained period of activity. Namely, we calculated the number of counts per bout of activity, the duration of activity bouts, the number of activity bouts per day and the peak rate of activity (defined as the peak number of counts per min).

### General Home Cage Activity and Immobility-Based Sleep

After the 2-week acclimatization period in LD, post-light disruption, mice were analyzed for circadian activity using the COMPASS passive infrared system as previously described (Brown et al., 2016, 2020). Briefly, a passive infrared sensor placed 220 mm above the cage floor monitored the animal’s activity. Activity within the PIR field was recorded in 10 s bins before re-binning for further analysis by custom Python scripts. Circadian analysis was performed by exporting activity data as AWD files for use in Clocklab (Actimetrics). Immobility-defined sleep analysis was performed by defining periods of 40 s or more as sleep, which has previously been validated against EEG-scored sleep (Brown et al., 2016).

### Body Temperature Rhythms

Body temperature rhythms were analyzed using near-infrared thermography, as previously described (van der Vinne et al., 2020).

### Light Input Pathways

#### Light-Induced cFos Expression

Mice were entrained to a 12:12 LD cycle for at least 14 days. On the day prior to the light pulse, the mice were released into DD. The following day, the mice were subjected to a 15-min, 300 lux light pulse at CT14. Ninety minutes after the end of the light pulse, the mice were sacrificed, and their brains were fixed with 4% paraformaldehyde. Sham pulsed mice, which were not subjected to a light pulse, were also treated in the same way. Using a vibratome (Leica), 35 μm coronal sections were taken through the SCN of each brain and placed in PBS. Sections were blocked in 2% goat serum in PBS for 1 h, before overnight incubation with primary antibody (rabbit-anti-cFos primary antibody (Abcam, AB222699) at 1:200 dilution) at 4*^o^*C. Following this, sections were washed in PBS before incubation for 1 h in AlexaFluor-488 conjugated donkey anti-rabbit secondary antibody (Thermo Fisher Scientific, A11070) diluted to 1:500 in PBS. Finally, sections were washed in PBS before mounting on slides. The labeled SCN sections were visualized using a Zeiss Axio-Examiner 710 NLO multi-photon microscope and images captured using Zen software (Zeiss). At least three SCN-containing images per animal were captured and used for counting of immunolabeled cells.

#### Pupillometry

Mice were tested between Zeitgeber Time (ZT) 4 and 8 and were dark-adapted for 1--2 h prior to testing. Animals were gently restrained (‘‘scruffed’’) upon a platform, positioned so that one eye could receive light from an LED source and the pupil response in the contralateral eye recorded by an IR-sensitive CCD camera (Prosilica, GC1020). Monochromatic (470 nm) light was delivered using an LED source (Luxeon Rebel LED assembly, SR-01-B0040) and pupillary light responses of the other eye was recorded. Initially, responses were recorded under bright light (14.6 log quanta/cm^2^/s). Lower irradiances (12.9, 11.6, 10.5 and 9.3 log quanta/cm^2^/s) were tested by positioning corresponding neutral density filters into the light path. The irradiances were measured and verified with a radiometrically calibrated spectrophotometer (Ocean Optics, United Kingdom). Consensual pupil responses were recorded using custom-made pupillometry software (Pupil6.3.vi running in LabView), capturing images at 10 frames per second (fps) in light, 5 fps in dark. The durations of recordings and stimuli were as follows: 2s in dark (pre-stimulus), 10s of light (stimulus) followed by 17s of dark (post-stimulus, recovery). Images were analyzed with ImageJ,^1^ allowing pupil area measurement for each image. In some cases, the pupil was not entirely visible, due to whiskers cutting the pupil area; this prevented the use of the automatic analysis. For these cases, pupil diameter was manually analyzed using ImageJ. The responsive pupil areas or diameter were calculated as a percentage of the baseline pupil area or diameter (when fully dilated under dark).

### Suprachiasmatic Nucleus Bioluminescence

Sixteen weeks after the recovery LD period brains were dissected from PER2::LUC knockin mice, mounted and cut into coronal slices (300 μm thick) on a vibrating blade microtome (Leica) in ice-cold HBSS supplemented with 100 U/ml penicillin/streptomycin, 10 mM HEPES, and 4.5 mM sodium bicarbonate. For each brain, the appropriate section containing the SCN was identified and the SCN area was dissected. SCNs were transferred to a Millicell cell culture membrane insert (PICM ORG 50, Millipore) with 1.2 mL of recording medium (DMEM (Invitrogen), supplemented with 10 mM HEPES (pH 7.2), 2% B27 (Invitrogen), 25 units/ml penicillin, 25 μg/ml streptomycin, and 0.1 mM luciferin). Individual tissue cultures were sealed in 35-mm Petri dishes with a coverslip using vacuum grease. Bioluminescence recordings from dissected SCNs were taken at 6 min intervals by a 32-channel/4-photomultiplier tube-containing LumiCycle (Actimetrics, IL, United States) apparatus, in a non-humidified, light-tight incubator maintained at 36°C. Data were analyzed on the Lumicycle Analysis software (Actimetrics, IL, United States). For the analysis, the first day of recordings were omitted as an “acclimatization day,” after which five consecutive cycles of bioluminescence were used for data analysis, to ascertain period and damping rate; only the first cycle was used to assess the acrophase of the rhythm.

### Non-circadian Behaviors

All non-circadian behavioral tests were performed between ZT1-4 under dim light (10 lux), to avoid masking and anxiogenic effects of light.

#### Rotarod

The rotarod test was used to assess motor function (Jones and Roberts, 1968). For the habituation phase (day 1), the rotarod (solid cylindrical rotating rod; Bioseb, Valbonne, France) rotated at a constant speed of 5 rotations per minute (rpm) for two 3-min-long trials, and 10 rpm for two 3-min-long trials. If mice fell off the rotarod during a habituation trial, they were put back on the rotarod until the timer ran out. A rest period of at least 15 min in the home cage was allotted to each mouse between trials. On the test day (day 2), the rotarod accelerated at a constant rate: from 4 to 40 rpm, over a 5-min span (acceleration = 7.2 rpm^2^). When the mouse fell off the cylinder, they would land onto a press plate located 20 cm below the rod. The amount of time that the mice stayed on the rotarod was recorded. This test was performed 3 times and mice were placed in their home cage between trials. Mice were excluded from the test if they did not attempt to walk on the rotarod and fell immediately.

#### Grip Strength Test

Motor function was also assessed using the grip strength test (Ge et al., 2016). Using only their forelimbs, mice were made to grasp a grid that was connected to a sensor. The mice were then gently pulled away from the grid using a horizontal motion, until the mice released the grid. Three such trials were carried out in succession. The maximal forelimb force of grip was measured for each trial and the average force was normalized for body weight.

#### Open Field Test

The open field test assesses spontaneous locomotor activity (Denenberg, 1969). The VersaMax Legacy Open Field setup (AccuScan Instruments, Inc., Columbus, OH, United States) was used. This system consists of acrylic chambers (*L* × *W* × *H* = 17.5 cm × 10 cm × 26 cm) equipped with infrared sensors to monitor locomotion. The center of each box was illuminated at appropriately 10 lux by cool white fluorescent room lighting. Mice were left in the boxes for 50 min to freely explore the chamber. Data were collected using the Versamax Software (version 4.0, 2004; AccuScan Instruments, Inc.). Measures such as total distance traveled and thigmotaxis, defined as the ratio of time spent in the margin (which represent 60% of the total area of the apparatus) over time spent in the center (which represents 40% of the total area of the apparatus), were analyzed.

#### Elevated Plus Maze

The elevated plus maze test was used to analyze anxiety-like behavior (Walf and Frye, 2007). The apparatus (made of wood, painted black, overlaid with gray mats) consists of a plus-shaped maze with two closed and two open arms, each 50 cm × 5 cm in size, raised 70 cm above the ground. The closed arms are enclosed by 10 cm high walls, while the open arms are not. Mice were placed in the center of the maze facing an open arm (10 lux from cool white fluorescent room lighting). Mice could freely explore the maze for 5 min, after which they were returned to their home cages. The session was recorded with a video camera positioned above the apparatus, and the recordings were scored for time spent in the open compared to the closed arms. A percentage of time spent in open arms was then calculated by dividing time spent in open arms by total time spent in both open and closed arms, multiplied by 100.

#### Morris Water Maze

Spatial learning and memory were assessed using the Morris water maze test (Vorhees and Williams, 2006; Barnhart et al., 2015). The maze apparatus consists of a circular swimming pool (diameter: 150 cm) filled with opaque water. Submerged 1 cm below the surface of the water, a platform (area: 225 cm^2^) or “escape area” was present. Four spatial cues were placed equidistantly around the pool on the pool walls to allow the mice to learn the location of the platform. The center of the maze had a light intensity of 50 lux. In the first 4 days of the experiment (acquisition trials), the mice were trained to locate the hidden platform. Each acquisition day consisted of 4 trials (with an inter-trial interval of at least 30 min), with the mouse beginning at a different location in the pool each time. The trial ended when the mouse located the hidden platform. The time taken to reach the platform was recorded at every trial to assess the speed of learning over the 4 days. On the 5th day of the experiment (the “probe trial”), the hidden platform was removed, and the time spent in each quadrant of the pool was recorded for 60 s. Lastly, in a “cue trial,” the platform was placed in a new location, to verify that the visual and motor systems were intact in the mice (Clapcote et al., 2005; Cendelin and Tichanek, 2020).

#### Prepulse Inhibition of Acoustic Startle

The prepulse inhibition (PPI) of acoustic startle reflex is a measure of sensory-motor gating (Niu et al., 2015; Swerdlow et al., 2016). Testing was carried out using the SR-Lab software connected to sound attenuating chambers equipped with plexiglass animal enclosure tubes (San Diego Instruments, San Diego, CA, United States). The chambers are ventilated by an electrical fan that produces a constant 70 dB background noise. Speakers positioned directly above the enclosure present tone pulses of differing loudness and the startle of the mouse is recorded by an accelerometer attached to the base of the enclosure. Within the experiment, following a 5-min acclimatization period in the tube, there were 3 phases to the paradigm. In the first and third phases, 6 startle pulses of 120 dB loudness and lasting for 30 ms each, were administered. In the second phase, 38 trials were administered. The first 8 trials were pulse only (startle only) trials. In the next 30 trials, the mouse received 30 ms prepulses of 0- (pulse alone), 6-, 9-, 12-, or 15-dB intensity above the background, 100 ms prior to the actual 120 dB pulse. These prepulses were randomly and equally varied across the 30 trials spaced 15 ms apart, with 6 of each of the prepulse trials presented to the mice. The average amplitude of startle in the last 15 startle-only trials is the baseline startle. %PPI was calculated as: 100 - [(startle response for prepulse followed by pulse trials/startle response for pulse alone trials) × 100].

### Dendritic Spine Analysis

Golgi-Cox neuronal staining was performed using the FD Rapid GolgiStain Kit (FD NeuroTechnologies Inc., United States) according to the manufacturer’s instructions. Sections (100 μm) were prepared using a vibratome, mounted on charged slides, cleared in Histo-Clear (National Diagnostics, United Kingdom), and cover-slipped. Neurons were viewed on an AxioObserver Z1 (Zeiss) microscope. Z-stack images were processed using extended depth of focus and Zen software (Zeiss). Visualization was carried out and measurements were made using ImageJ^1^. The number and type of spines on each neurite were counted (Risher et al., 2014). Cortical neuron images were captured from layers 2–3 of the cortex and hippocampal neuron images were captured from the CA1 region. These regions were selected as they were the regions in which the greatest clarity of staining was observed. At least 50 neurites per region per animal were analyzed.

### Clinical Chemistry

Fourteen weeks after the recovery LD period, trunk blood was collected from mice in lithium heparin coated tubes, on ice. The blood was then centrifuged, and the serum was stored at –70^°^C. Clinical chemistry analysis was performed by the MRC Harwell Clinical Chemistry service, using a Beckman Coulter AU680 clinical chemistry analyzer.

### Statistics

The data were plotted and analyzed on GraphPad Prism version 9. Data were analyzed for homogeneity of variances (using Bartlett’s test) and normality of data (using Shapiro-Wilk test). If data passed normality and homogeneity of variances, one-way, two-way or repeated-measures ANOVAs were used to analyze data between groups (LD, DLAN, SJ), as appropriate to the data. All pairwise comparisons used the Benjamini-Hochberg corrections. Welch’s correction was applied if variances were unequal between groups and Kruskal-Wallis non-parametric test was conducted if data did not pass normality test. Differences were considered significant if *p* < 0.05.

## Results

### Activity Rhythms of Grouped Mice Over the Year in Different Lighting Conditions

**FIGURE 2:**
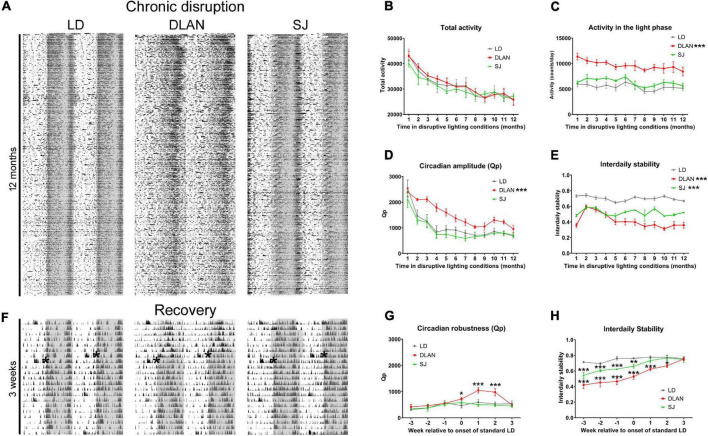
Passive infrared recordings (PIR) monitoring during the 12-month lighting disruption period. **(A)** Representative actograms of group-housed mouse activity throughout the 12-month period of disruptive lighting. **(B–E)** Quantification of the PIR data over the 12 months of recording: total activity levels **(B)**, activity in the light phase **(C)**, circadian amplitude (Qp value) **(D)**, and interdaily stability **(E)**. **(F)** Representative actograms of the recovery period following the 12-month period of disruptive lighting. Actograms show the final week of activity under disruptive conditions and the following 2 weeks after return to standard LD conditions. Asterisks in the actograms of panel F denote the onset of standard LD conditions. **(G,H)** Quantification of PIR data of the recovery period: Qp **(G)** and interdaily stability **(H)**. Data is presented as mean ± SEM. Repeated measures ANOVA (with Welch correction), groups: LD, DLAN, SJ. *Post-hoc* tests conducted with Benjamini-Hochberg corrections. ***p < 0.001, **p < 0.01, *p < 0.05.

To establish how chronic exposure to abnormal light cycles modulates activity rhythms during the 12-month period of disruption (Figure 1), after a 2-week recovery period, the activity of group-housed mice (6 cages per lighting condition, 5 mice per cage) was monitored by passive infrared sensors (PIR). Representative actograms of these recordings are shown in Figure 2A (close-up views of recordings after 1, 6 and 12 months of disruptive lighting are shown in Supplementary Figure 1A). The data extracted from this activity monitoring was analyzed using repeated measures ANOVAs to determine whether there was a significant effect of lighting condition alone or a significant effect of the interaction between the lighting condition and the time (in months) in which animals had been subjected to disruption. Here we found no significant differences in the total daily activity levels of mice with respect to lighting condition [*F*_(2,__15)_ = 0.25; *p* = 0.78] or the interaction between lighting condition and time [*F*_(22,__165)_ = 0.7; *p* = 0.83], suggesting that total daily animal activity was unaffected by our chronic disruption conditions (Figure 2B). However, analysis of light phase activity indicated a significant difference between lighting conditions [*F*_(2,__15)_ = 38.32; *p* = 0.0001], but no interaction between lighting condition and time [*F*_(22,__165)_ = 0.83; *p* = 0.674; Figure 2C]. Pairwise comparisons demonstrated that mice under the DLAN condition showed significantly higher light phase activity than either control or SJ (*p* < 0.0001 for both DLAN vs. LD and DLAN vs. SJ) but showed no differences between LD and SJ (*p* = 0.145). Given that the different lighting conditions resulted in differences in total light exposure (animals under LD have 84 h per week of light exposure, whereas animals under SJ have 75 h per week), we analyzed light phase activity as average light phase activity per hour in order to normalize for this difference in light exposure (Supplementary Figure 1B). This analysis indicated a significant difference between lighting conditions [*F*_(2,__15)_ = 33.06; *p* = 0.0001], but no interaction between lighting condition and time [*F*_(22,__165)_ = 0.87; *p* = 0.636]. Pairwise comparisons demonstrated that mice under the DLAN condition showed significantly higher average hourly light phase activity than either control or SJ (*p* < 0.0001 for both DLAN vs. LD and DLAN vs. SJ) and an increase in average hourly light phase activity in SJ compared to controls (*p* = 0.0147). Repeated measures ANOVA analysis of the robustness of circadian activity rhythms (Qp value, as measured by Lomb-Scargle periodogram power) highlighted a significant difference at both the levels of lighting condition alone [*F*_(2,__15)_ = 8; *p* = 0.0043] and upon the interaction between lighting condition and time [*F*_(22,__165)_ = 2.74; *p* = 0.0001; Figure 2D]. Pairwise comparisons demonstrated that the Qp of mice under the DLAN condition significantly differed from that of the other two conditions (*p* < 0.0001 DLAN vs. LD and *p* = 0.004 for DLAN vs. SJ) but did not between LD and SJ (*p* = 0.645), suggesting that DLAN had a significant impact upon the robustness of activity rhythms. To investigate this further we conducted repeated measures ANOVAs on the interdaily stability (IS) and the intradaily variability (IV) of the activity rhythms. Here we found that the IS of our treatment groups was significantly affected by both the levels of lighting condition alone [*F*_(2,__15)_ = 60.91; *p* = 0.0001] and upon the interaction between lighting condition and time [*F*_(22,__165)_ = 11.8; *p* = 0.0001; Figure 2E]. Pairwise comparisons showed that all lighting conditions significantly differed from each other in IS measures (*p* < 0.0001 for all comparisons). By contrast the IV of activity rhythms was unaffected by the lighting conditions [*F*_(2,__15)_ = 0.83; *p* = 0.455] or the interaction between lighting condition and time [*F*_(22,__165)_ = 1.12; *p* = 0.33].

The above analysis suggested that the age-related change of both Qp and IS differs during our disruptive lighting conditions. To identify when during the disruption period these differences occur, we performed further repeated measures ANOVAs of the Qp and IS data, incorporating a pairwise analysis of monthly time bins. For Qp values, we found no differences between LD and SJ groups. However, DLAN mice significantly differed from both LD and SJ groups during the initial 6 months of exposure to the disruptive conditions (*p* < 0.05 for LD vs. DLAN and DLAN vs. SJ during months 2–6 of lighting exposure). There were no significant differences found in the subsequent months. For IS values, the majority of pairwise comparisons were significantly different (*p* < 0.05) for any combination of lighting conditions, across different months. The exception to this was comparisons between DLAN and SJ at months 2–5 of exposure (*p* > 0.05 for these time points).

Finally, visual inspection of the Qp and IS data suggested that mice in different lighting conditions acclimatized to the disruption at different rates. To test this hypothesis, we performed one-way ANOVAs on the monthly Qp and IS data from each lighting condition, using Dunnett’s multiple procedure to establish which time point significantly differs from the final (12-months) time point. In this analysis, both the LD and SJ groups showed the same significance pattern, in which Qp data was only significantly different in the first month of recordings (Qp for month 1 vs. month 12, *p* < 0.05) and IS showed no significant differences in any of the comparisons made. This suggested that animals placed into SJ maintained a stable IS for the duration of the exposure to the disruption, whereas the Qp of their activity rhythms decreased for the first month of exposure, before stabilizing for the remaining duration of the disruption. The fact that these patterns of change are the same as for animals in the control LD condition implies that this initial change in Qp is a result of healthy aging rather than the impact of SJ light cycles. In contrast, when we compared the final month of the year of light disruption to each of the previous 11 months, the Qp of mice housed in DLAN conditions significantly differed from the months 1 through to 5 (Qp for month 1–5 vs. month 12, *p* < 0.05) before dropping beneath significance for the remainder of the disruption period (Qp for month 6–11 vs. month 12, *p* > 0.05). Similar analysis demonstrated that the IS for DLAN mice significantly differed from the final month’s disruption at months 2 and 3 (Qp for month 2 and 3 vs. month 12, *p* < 0.05). Taken together this data suggests that mice under DLAN take longer to acclimatize to abnormal lighting conditions than mice in LD or SJ.

### Recovery From Chronic Lighting Disruption

Following the chronic period of disruption, mice were returned to a standard 12:12 LD cycle. This was aimed to allow for recovery from the irregular lighting conditions, so that we could assay the long-term consequences of such lighting conditions, rather than the confounding acute effects on our measures. To establish how the mice recovered from this prolonged period of abnormal lighting, we continued activity monitoring during this recovery phase (Figure 2F). Analysis of the Qp and the IS during recovery demonstrated that mice under DLAN conditions re-established normal rhythms by 3 weeks of standard light cycles [repeated measures ANOVA, *F*_(10,__60)_ = 16.84; pairwise comparisons *p* < 0.05 by week 3 following LD cycle for both Qp and IS]. In contrast, mice under the SJ condition re-established normal rhythms within a week of the establishment of standard lighting conditions [repeated measures ANOVA, *F*_(10,__60)_ = 16.84; pairwise comparisons *p* < 0.05 by week 1 following LD cycle for IS and no significant differences prior or post standard conditions for Qp; Figures 2G,H].

### Wheel Running Activity

**FIGURE 3:**
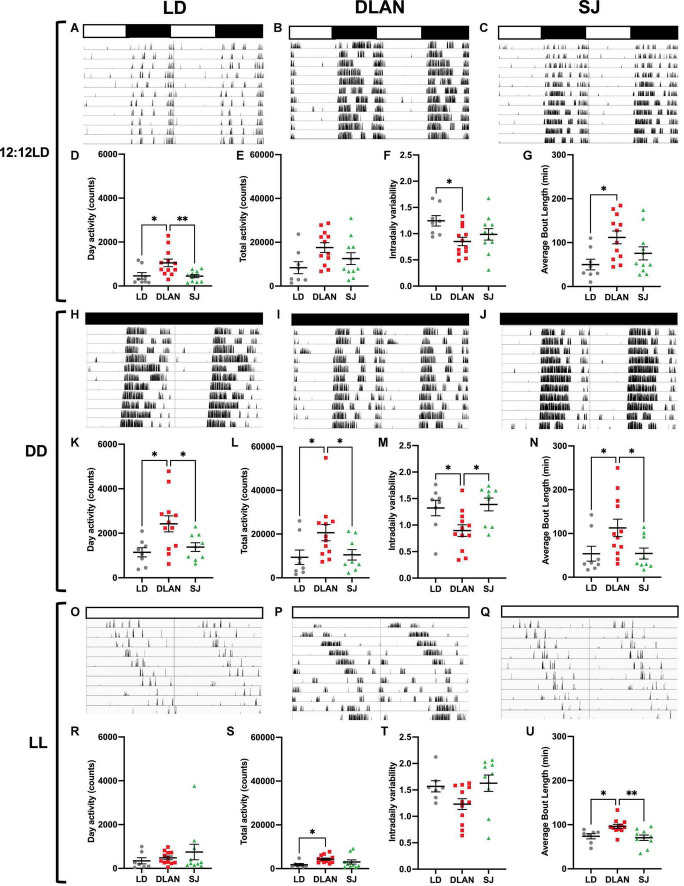
Wheel running activity analysis under 12:12 LD, DD and LL, following the end of the 12-month lighting disruption period. Representative actograms for each lighting condition (listed vertically: 12:12LD, DD, LL) are shown for each group: LD **(A,H,O)**, DLAN **(B,I,P)**, and SJ **(C,J,Q)** (see Figure 1 for a description of the three groups). Data are double plotted to facilitate visualization. Variables were analyzed in the last 10 days of each condition, including (subjective) day activity counts **(D,K,R)**, total activity counts **(E,L,S)**, intradaily variability **(F,M,T)** and average bout length **(G,N,U)**. Individual data points represent independent mice and group means ± SEM are presented. One-way ANOVA (with Welch correction, or Kruskal-Wallis test when appropriate), groups: LD, DLAN, SJ. *Post-hoc* tests conducted with Benjamini-Hochberg corrections when appropriate. Supplementary Table 1 lists the mean ± SEM of all analyzed parameters. ^**^p < 0.01, *p < 0.05.

We explored if chronic exposure to abnormal lighting had long term effects on circadian locomotor activity rhythms using voluntary running wheel activity, measured after the end of the year in these conditions (Figure 1). Running wheel activity of singly housed mice was recorded in three different lighting conditions: 12:12LD cycles (to assess response to an entraining stimulus), constant darkness (DD; to assess activity in free running conditions) and constant light (LL; a condition which weakens the network of the central clock, the SCN; Ohta et al., 2005). Representative actograms for each lighting condition (listed vertically: 12:12LD, DD, LL) were shown for each group: LD (Figures 3A,H,O), DLAN (Figures 3B,I,P), and SJ (Figures 3C,J,Q). Supplementary Table 1 lists the mean ± SEM of all analyzed parameters.

Groups exhibited similar values for period (h) (12:12LD: *p* = 0.9999, DD: *p*_*adj*_ = 0.8441, LL: *p*_*adj*_ = 0.8650) and alpha (h), defined by the duration of the active phase, under each lighting condition (12:12LD: *p* = 0.3023, DD: *p* = 0.4929, *LL: p_adj_* = 0.8365; Supplementary Table 1). Interestingly, we report a significant effect of group for (subjective) day activity under 12:12LD [Kruskal-Wallis, *H*_(2)_ = 9.542; *p* = 0.0085; Figure 3D] and under DD [one-way ANOVA, *F*_(2, 26)_ = 5.623; *p* = 0.0093; Figure 3K] but not LL (*p*_*adj*_ = 0.3757; Figure 3R). *Post hoc* analyses revealed that under both 12:12LD and DD, the DLAN group had significantly more (subjective) day activity than the LD group (12:12LD: *p*_*adj*_ = 0.0136, DD: *p*_*adj*_ = 0.0163) and the SJ group (12:12LD: *p*_*adj*_ = 0.0136, DD: *p*_*adj*_ = 0.0251). When observing (subjective) night activity, we observed a significant effect of group under DD [Kruskal-Wallis, *H*_(2)_ = 7.356; *p* = 0.0253] and LL [Kruskal-Wallis, *H*_(2)_ = 10.68; *p* = 0.0048] but not 12:12LD [one-way ANOVA, *F*_(2, 28)_ = 2.748; *p* = 0.0813; Supplementary Table 1]. *Post hoc* analyses revealed that under both 12:12LD and DD, the DLAN group had significantly more (subjective) night activity than both the LD group (12:12LD: *p*_*adj*_ = 0.0436, DD: *p*_*adj*_ = 0.0091) and the SJ group (12:12LD: *p*_*adj*_ = 0.0436, DD: *p*_*adj*_ = 0.0203). Finally, for total activity, we noted a significant effect of group under DD [Kruskal-Wallis, *H*_(2)_ = 7.387; *p* = 0.0249], where DLAN mice had more total activity than LD (*p*_*adj*_ = 0.0470) and SJ (*p*_*adj*_ = 0.0470; Figure 3L) and LL [Kruskal-Wallis, *H*_(2)_ = 9.167; *p* = 0.0102; Figure 3S] where DLAN had more totally activity than LD (*p*_*adj*_ = 0.0043). No differences were observed in total activity under 12:12LD [one-way ANOVA, *F*_(2, 28)_ = 3.117; *p* = 0.0599; Figure 3E]. No significant differences were seen in percent (subjective) day activity between groups in any of the lighting conditions (12:12LD: *p* = 0.4914, DD: *p* = 0.9276, LL: *p*_*adj*_ = 0.1225; Supplementary Table 1). Overall, we observed patterns of increased activity counts in the DLAN group compared to other groups.

There was a significant effect of group for relative amplitude (RA) under DD [Kruskal-Wallis, *H*_(2)_ = 6.052; *p* = 0.0485], with no significant *post hoc* analyses, and LL [Kruskal-Wallis, H_(2)_ = 6.406; *p* = 0.0406] where DLAN had higher relative amplitude scores than SJ (*p*_*adj*_ = 0.0367; Supplementary Table 1). We also found a significant effect of group in intradaily variability (IV) under 12:12LD [one-way ANOVA, *F*_(2, 28)_ = 3.912; *p* = 0.0317; Figure 3F] and DD [Figure 3M; one-way ANOVA, *F*_(2, 26)_ = 5.147; *p* = 0.0131] but not in LL [one-way ANOVA, *F*_(2, 26)_ = 3.176; *p* = 0.0584; Figure 3T] *Post hoc* comparisons revealed that the LD group had higher IV than DLAN under 12:12LD (*p* = 0.0281). Under DD, the DLAN group had significantly lower IV than SJ (*p* = 0.0218) and LD (*p* = 0.0334). No significant differences were observed in IS between groups (Supplementary Table 1).

Interestingly, there was a significant group effect for average bout length under all three lighting conditions: 12:12LD [one-way ANOVA, *F*_(2, 28)_ = 4.44; *p* = 0.0211; Figure 3G], DD [Kruskal-Wallis, *H*_(2)_ = 7.069; *p* = 0.0292; Figure 3N] and LL [one-way ANOVA, *F*_(2, 26)_ = 7.317; *p* = 0.0030; Figure 3U]. Pairwise comparisons revealed that DLAN had increased bout lengths compared to LD under 12:12LD (*p* = 0.0212), DD (*p*_*adj*_ = 0.0397) and LL (*p* = 0.0151), and DLAN had increased bout length compared to SJ only under DD (*p*_*adj*_ = 0.0397) and LL (*p* = 0.0046).

There were also significant differences across lighting conditions for average counts per bout [12:12LD: Kruskal-Wallis, *H*_(2)_ = 7.941; *p* = 0.0189, DD: Kruskal-Wallis, *H*_(2)_ = 8.380; *p* = 0.0151 and LL; Kruskal-Wallis, H_(2)_ = 9.472; *p* = 0.0088; Supplementary Table 1]. *Post-hoc* revealed that DLAN had an increased average counts per bout than LD (12:12LD: *p*_*adj*_ = 0.0163; DD: *p*_*adj*_ = 0.0321; LL: *p*_*adj*_ = 0.0166) and SJ (DD: *p*_*adj*_ = 0.0321; LL: *p*_*adj*_ = 0.0282).

Finally, there was a significant effect of group for average peak rate under 12:12LD [one-way ANOVA, *F*_(2, 28)_ = 5.229; *p* = 0.0118], with DLAN having an increased peak rate compared to LD (*p*_*adj*_ = 0.0106; Supplementary Table 1). No significant differences were observed in the number of bouts in any lighting conditions [12:12LD: *p* = 0.1586, DD: *p* = 0.0586, LL: *p* = 0.1126; Supplementary Table 1].

### General Home Cage Activity and Immobility-Based Sleep

**FIGURE 4:**
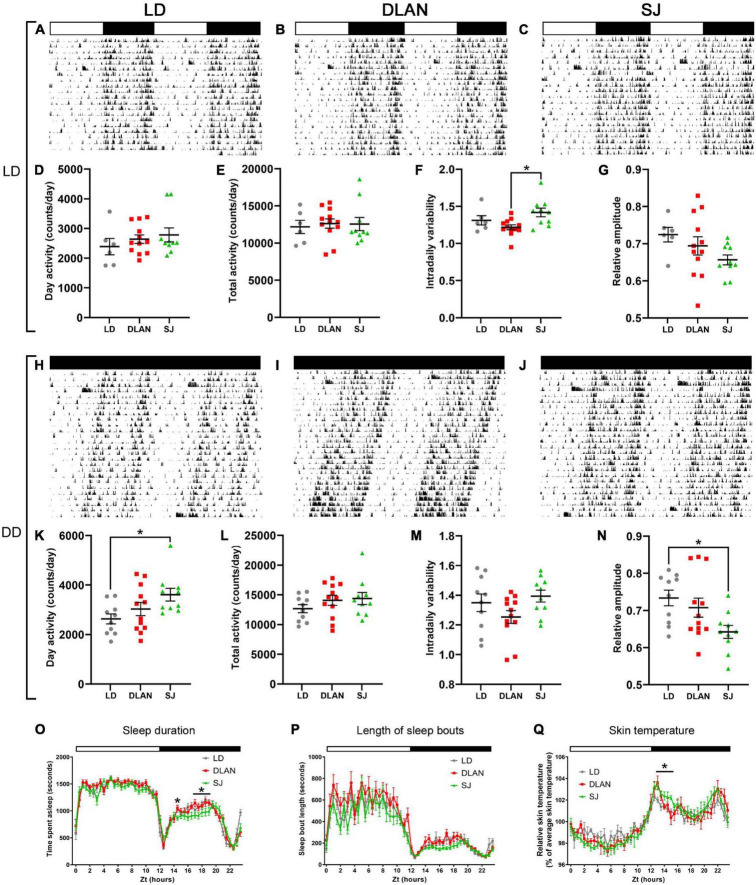
Passive infrared recordings (PIR) of single-housed mice, following the end of the 12-month lighting disruption period. Representative actograms for each lighting condition (listed vertically: 12:12LD, DD) are shown for each group: LD **(A,H)**, DLAN **(B,I)**, and SJ **(C,J)** (see Figure 1 for a description of the three groups). Data are double plotted to facilitate visualization. Variables were analyzed over a period of 20 days in each condition, including activity during the (subjective) day **(D–K)**, total daily activity **(E)**, free-running period **(L)**, intradaily variability **(F–M)** and relative amplitude **(G–N)**. **(O,P)** Amount of time spent in immobility-defined sleep (O) and the length of immobility-defined sleep bouts **(P)** under LD **(O)**, for the three groups. **(Q)** Skin temperature under LD, for the three groups. **(D–G,K–N)** One-way ANOVA (with Welch correction, or Kruskal-Wallis test when appropriate), groups: LD, DLAN, SJ. *Post-hoc* tests conducted with Benjamini-Hochberg corrections when appropriate. Supplementary Table 2 lists the mean ± SEM of all analyzed parameters. **(O–Q)** Repeated measures ANOVA (with Welch correction), groups: LD, DLAN, SJ. *Post-hoc* tests conducted with Benjamini-Hochberg corrections. *p < 0.05.

To complement the circadian running wheel analysis, we used passive infrared monitoring on a distinct cohort of animals to screen for long-term effects of chronic disruption upon circadian activity in both 12:12 LD cycles and DD. The complete set of analyzed parameters for this can be found in Supplementary Table 2. Under 12:12 LD (Figures 4A–C), mice showed no significant group differences in levels of activity (e.g., activity in the light phase Figure 4D or total daily activity Figure 4E). However, we found a significant effect in IV [one-way ANOVA, *F*_(2,__25)_ = 4.76; *p* = 0.018] (Figure 4F). Pairwise comparisons demonstrated that this difference was between the DLAN and SJ groups (*p* = 0.015), with no significant differences found between either disrupted condition and the LD group. Other circadian parameters (e.g., relative amplitude Figure 4G) were unchanged in the abnormal lighting groups under 12:12 LD cycles (Supplementary Table 2).

In contrast, in DD conditions (Figures 4H–J) our analysis identified a significant difference in the amount of activity in the subjective day (rest phase) [one-way ANOVA, *F*_(2,__29)_ = 3.71; *p* = 0.037; Figure 4K]. Pairwise comparisons indicated that this difference was due to an increase in the amount of activity in the SJ group when compared to LD controls (*p* = 0.034). We also identified a significant difference in circadian period between the groups in DD [one-way ANOVA, *F*_(2,__29)_ = 5.37; *p* = 0.01; Figure 4L]. Pairwise comparisons showed a shortening of period in the DLAN group when compared to LD and SJ groups (vs. LD *p* = 0.015; vs. SJ *p* = 0.015). In contrast to under 12:12 LD cycles, there were no differences between groups in IV (Figure 4M). However, there was a significant difference between groups in RA [one-way ANOVA, *F*_(2,__29)_ = 4.19; *p* = 0.025; Figure 4N], with a reduction in the SJ group relative to the LD group in pairwise comparisons (*p* = 0.027). No other significant changes in circadian parameters were found when the mice were in DD.

In addition to activity analysis, passive infrared sensors can also monitor immobility-defined sleep based upon extended immobility (Brown et al., 2020). Here we found no changes in the total amount of time spent asleep in mice from either abnormal lighting condition. However, SJ mice showed a significant change in their sleep patterns across the day [repeat measures ANOVA, interaction factors “lighting condition × time spent asleep”; *F*_(47,__1034)_ = 2.62; *p* = 0.0001; Figure 4O]. Pairwise comparisons demonstrated that the majority of this difference was due to a reduction in the duration of sleep in mice from the SJ group at the mid-dark phase (significance between control and SJ group was *p* < 0.05 for ZT 17–19). Mice from DLAN conditions showed no significant differences in sleep pattern [repeated measures ANOVA, interaction factors “lighting condition × time spent asleep”; *F*_(47,__1034)_ = 1.3; *p* = 0.0852]. Additionally, in order to assess sleep fragmentation, we measured the average length of the sleep bouts for these mice. Here we found no significant differences in either lighting conditions (Figure 4P).

Finally, in addition to activity and sleep-based measures of rhythmicity, we also used near-infrared thermography to screen for circadian differences in skin temperature when mice were in a 12:12 LD cycles (van der Vinne et al., 2020; Figure 4Q). Mice of the SJ group showed a significant change in their temperature cycles compared to the LD group [repeated measures ANOVA, interaction factors “lighting condition × relative temperature”; *F*_(47,__470)_ = 1.84; *p* = 0.009]. This difference was mainly due to an elevation of skin temperature in SJ mice in the early dark phase (LD vs. SJ *p* < 0.05 from ZT 13 to 14.5).

### Light Input Pathways

Light input undergoes a variety of functional changes during healthy aging (Semo et al., 2003; Lupi et al., 2012). Our circadian analyses found no evidence that aberrant lighting conditions exacerbate this decline: we found no difference in entrainment to the 12:12 LD cycles in the abnormal lighting groups.

We analyzed retinal sensitivity to light input further by measuring the pupillary light response to a range of light intensities. There was no significant difference between groups for the IC_50_ of the response [one-way ANOVA, *F*_(2,__14)_ = 2.65; *p* = 0.106; Supplementary Figure 2A] or for the maximum pupil constriction across different light intensities [repeated measures ANOVA, interaction factors “lighting condition × pupil constriction”; *F*_(13,__37)_ = 1.53; *p* = 0.197; Supplementary Figure 2B].

Additionally, we analyzed light input into the SCN of the experimental mice by immunolabeling for cFos within the SCN, following a light pulse at CT14 (early night). Two-way ANOVA analysis demonstrated that while cFos staining was significantly increased by light pulse exposure compared to sham animals [*F*_(1,__18)_ = 1770; *p* < 0.0001], there was no significant differences in cFos staining between different lighting groups [*F*_(2,__18)_ = 0.4; *p* = 0.675] or in the interaction between light pulse exposure and disruptive light condition [*F*_(2,__18)_ = 0.41; *p* = 0.667; Supplementary Figure 2C]. Taken together these data suggest that light input pathways were unaffected in mice after chronic abnormal lighting conditions.

### Suprachiasmatic Nucleus Bioluminescence Rhythms

**FIGURE 5:**
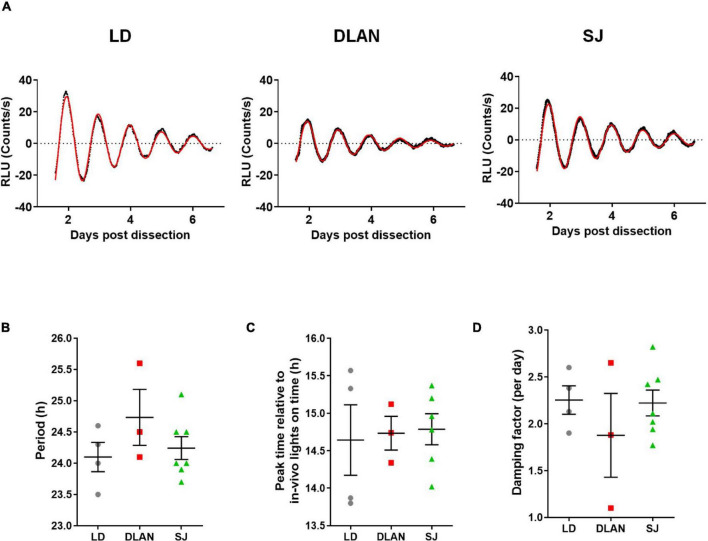
*Ex-vivo* bioluminescence of the SCN of mice, following the 12-month light disruption protocol. **(A)** Representative bioluminescence traces are shown for each of the experimental groups. The black line represents the actual data, and the red line represents the best fit line, using a damped sine fit. From these data, the period **(B)** acrophase **(C)** and damping rate **(D)** are shown. Individual data points represent independent SCNs and group means ± SEM are presented. One-way ANOVA (with Welch correction when appropriate), groups: LD, DLAN, SJ. *Post-hoc* tests conducted with Benjamini-Hochberg corrections when appropriate.

To establish the effect of chronic abnormal lighting conditions on the molecular rhythms of the central pacemaker of the SCN, we compared the bioluminescence rhythms of PER2::LUC reporter mice that went through the different lighting conditions (Figure 5A). We looked at three main measures of rhythmicity: period (the duration of each cycle of the rhythm), acrophase (the peak of the rhythm) and damping (the robustness and synchronization of the rhythms). We found no significant differences in any of the parameters analyzed [Period: one-way ANOVA, *F*_(2, 11)_ = 1.3; *p* = 0.3226; Acrophase: one-way ANOVA with Welch correction, *W*_(2, 5.4)_ = 0.04, *p* = 0.9613; Dampening rate: one-way ANOVA, *F*_(2, 11)_ = 0.729; *p* = 0.5043; Figures 5B–D], suggesting that the molecular clock of the SCN and the neuronal network within this tissue were not evidently affected by abnormal lighting.

### Tests of Motor Function and Coordination, Anxiety-Like Behavior, Cognitive Function, and Sensory Motor Gating

**FIGURE 6:**
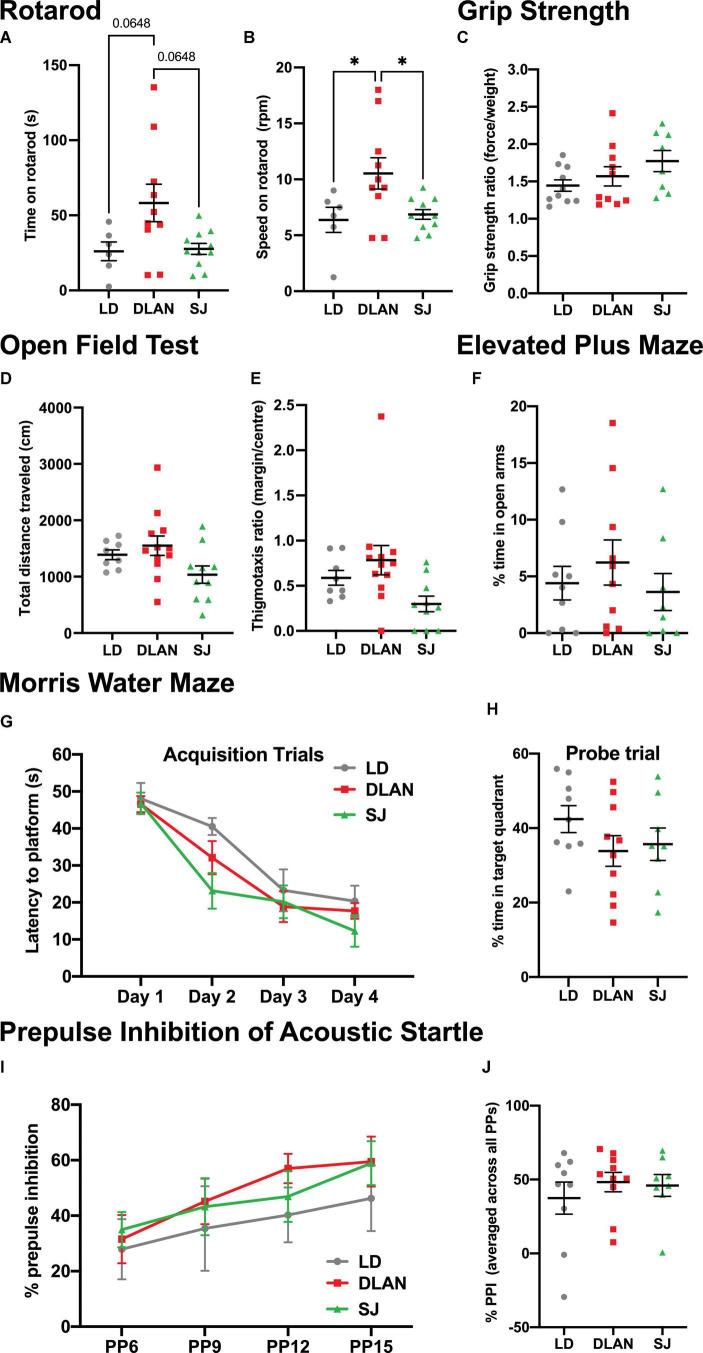
Chronic exposure to abnormal lighting conditions affects some measures of motor function, but not exploratory behavior, anxiety-like behavior, spatial learning and memory, and sensory motor gating. **(A–C)** Motor function was tested on the rotarod, where time on rotarod **(A)** and speed on rotarod **(B)** were measured. **(C)** Forelimb grip strength was also assessed as a measure of motor function. **(D–F)** Exploratory and anxiety-like behaviors were analyzed using the open field test, where total distance traveled **(D)** and thigmotaxis ratio **(E)** were assessed. The elevated plus maze test was also used to assess anxiety-like behavior, where percentage of time in open arms was measured **(F)**. **(G,H)** In the Morris Water Maze test, latency to platform on the acquisition trials was measured to assess spatial learning **(G)** and percent time to target quadrant on the probe trial was measured to assess spatial memory **(H)**. **(I,J)** For the prepulse inhibition of acoustic startle test, percent prepulse inhibition over the different prepulse intensities **(I)** and percent prepulse inhibition averaged over all prepulse intensities **(J)** were measured to assess sensory motor gating. Individual data points represent independent mice and group means ± SEM are presented. One-way ANOVA (with Welch correction, or Kruskal-Wallis test when appropriate), groups: LD, DLAN, SJ. *Post-hoc* tests conducted with Benjamini-Hochberg corrections when appropriate. *p < 0.05.

Deficits of motor function are known to occur in aged rodents (Serradj and Jamon, 2007). We tested whether chronic exposure to abnormal lighting conditions impacted this motor decline. We report a significant effect of group on the time spent on the rotarod [Kruskal-Wallis, *H*_(2)_ = 6.207; *p* = 0.0449], with a tendency for DLAN to spend more time on the rotarod than LD (*p*_*adj*_ = 0.0648) and SJ (*p*_*adj*_ = 0.0648) (Figure 6A). We also found a significant effect of group on speed achieved before mice fell off the rotarod [one-way ANOVA, *F*_(2, 15.73)_ = 4.784; *p* = 0.0178; Figure 6B], with mice of the DLAN group able to remain on the rotarod at faster speeds compared to LD (*p* = 0.0251) and SJ (*p* = 0.0251). Notably, however, groups performed similarly on a forelimb grip strength test, another measure of motor function [one-way ANOVA, *F*_(2, 25)_ = 1.891; *p* = 0.1719; Figure 6C].

Exploratory and anxiety-like behaviors were analyzed using the open field and elevated plus maze tests. We report no significant group differences in open field measures of total distance traveled [one-way ANOVA, *F*_(2, 27)_ = 2.997; *p* = 0.0667; Figure 6D] and thigmotaxis ratio [Brown-Forsythe ANOVA, *F*_(2, 14.96)_ = 1.211; *p* = 0.3254; Figure 6E]. Similarly, in the elevated plus maze test, we found no significant group differences in the percent time spent in the open arms [Kruskal-Wallis, *H*_(3)_ = 1.191; *p* = 0.5512; Figure 6F], suggesting that anxiety-like behavior is unaffected by chronic exposure to abnormal lighting conditions.

To test spatial learning and memory, the Morris Water Maze test was conducted. No significant differences were found between groups in acquisition trials [repeat measures ANOVA, interaction factors “lighting treatment × day”; *F*_(6,__72)_ = 1.279; *p* = 0.2967; Figure 6G], or in the probe trial [one-way ANOVA, *F*_(2, 24)_ = 1.279; *p* = 0.2967; Figure 6H], suggesting that spatial learning and memory was unaffected by chronic exposure to abnormal lighting conditions. Additionally, no differences were found in the cue trial [time to platform group means (seconds): LD = 16.9, DLAN = 21.4 and SJ = 27.6; one-way ANOVA, *F*_(2,__24)_ = 0.9719; *p* = 0.3928].

Prepulse inhibition (PPI) of acoustic startle was used to test sensory motor gating. All groups exhibited a similar percent PPI over the different prepulse tone intensities [repeat measures ANOVA, interaction factors “lighting treatment × prepulse”; *F*_(6,__72)_ = 0.4094; *p* = 0.870], and the percent PPI increased similarly for all experimental groups with increasing prepulse levels (*p* < 0.01) (Figure 6I). Finally, all experimental groups had similar precent PPI scores when data were averaged across all prepulse levels [Kruskal-Wallis, *H*_(3)_ = 0.5192; *p* = 0.7714; Figure 6J]. These data suggest that sensory motor gating was unaffected by chronic exposure to abnormal lighting conditions.

### Dendritic Spines in Cortex and Hippocampus

**FIGURE 7:**
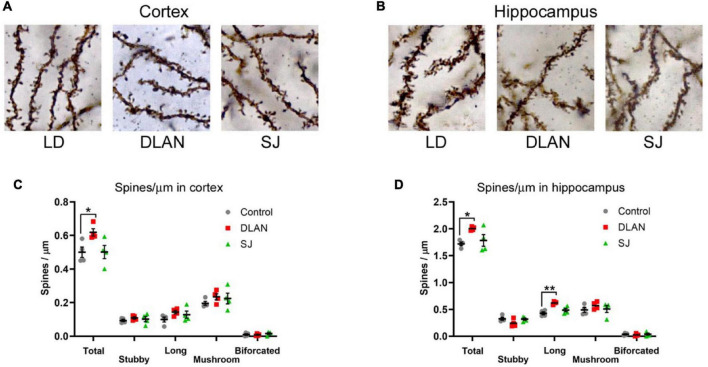
Dendritic spine analysis, following the end of the 12-month lighting disruption period. **(A,B)** Representative images of dendritic spine visualization in the cortex **(A)** and hippocampus **(B)** of mice from disruptive conditions (see Figure 1 for a description of the three groups). **(C,D)** Quantification of the density of dendritic spines in the cortex **(C)** and hippocampus **(D)**, classified by morphology, and total numbers. One-way ANOVA (with Welch correction, or Kruskal-Wallis test when appropriate), groups: LD, DLAN, SJ. *Post-hoc* tests conducted with Benjamini-Hochberg corrections when appropriate. **p < 0.01 and *p < 0.05.

Previous work has established that exposure to disruptive lighting can induce alterations in dendritic spines (Bedrosian et al., 2011; Karatsoreos et al., 2011; Aubrecht et al., 2013). To establish whether the phenotypes we observed in the mice might be paralleled by changes in the synapses in the brain, we performed spine count analysis on Golgi stained neurons from the cortex (Figure 7A) and the hippocampus (Figure 7B).

Analysis of the total number of dendritic spines in cortical sections demonstrated a significant difference between groups [one-way ANOVA, *F*_(2, 9)_ = 4.65; *p* = 0.041]. Pairwise comparisons indicated that this difference was due to a significant increase in spine numbers in the DLAN group compared to both the LD and SJ groups (*p* = 0.0412 for both). There was no significant difference between the LD and SJ groups (Figure 7C). The maturity of dendritic spines can be established through the analysis of their morphology, with different types of spines (stubby, long, mushroom or bifurcated) reflecting a continuum of spine maturity (Risher et al., 2014). However, we found no significant differences in any specific spine types in the cortex (Figure 7C).

We conducted a similar analysis in hippocampal sections, and also found a significant effect of group in total spine numbers [one-way ANOVA, *F*_(2, 9)_ = 5.14; *p* = 0.0325]. Pairwise comparisons indicated that this difference was due to a significant increase in spine numbers in the DLAN group compared to the LD group (*p* = 0.041), with the difference between the DLAN and SJ groups being near significant (*p* = 0.063). There was no significant difference between the LD and SJ groups (Figure 7D). Analysis of different spine types in the hippocampus demonstrated a significant difference in the numbers of long spines between the lighting groups [one-way ANOVA, *F*_(2, 9)_ = 14.71; *p* = 0.0015], due to an increase in spine numbers in the DLAN group compared to both the LD and SJ groups (*p* = 0.0015 and 0.0075, respectively).

### Clinical Chemistry

Changes in health are often associated with changes in a range of biomarkers in the blood. To establish whether chronic disruptive lighting conditions was associated with any such changes, clinical chemistry analysis was performed on serum samples from mice in the early light phase (ZT3) and in the early night (ZT15). A panel of clinical chemistry tests were used to identify changes in the activities of enzymes, alkaline phosphatase, alanine aminotransferase, amylase and lactate dehydrogenase and in the levels of albumin, calcium, chloride, cholesterol, potassium, sodium, proteins, triglycerides and urea. Following ANOVA analysis and multiple test corrections, we found no significant differences in any of the clinical chemistry tests performed, for either lighting condition at either time point (*p* > 0.05; Supplementary Figure 3), suggesting that chronic exposure to disruptive lighting has minimal effects on clinical chemistry biomarkers.

## Discussion

Mistimed or disruptive exposure to light has been demonstrated to negatively affect numerous aspects of physiology and behavior (Karatsoreos et al., 2011; Evans and Davidson, 2013). Our understanding of these impacts has been advanced using animal models which are first subjected to disruptive lighting environments and subsequently phenotyped to test the physiological or behavioral impact of the disruption. However, in a majority of studies, the disruption period is often of short duration (lasting from several weeks to a few months), whereas in humans, circadian disruption such as shift work or exposure to light at night can last for years. Here we present an analysis of the effects of long-term exposure to disruptive lighting conditions in mice. We subjected animals to one of two different disruptive light cycles for a period of 1 year: Dim Light at Night (DLAN) or Social Jetlag (SJ). For the latter, we acknowledge that social jetlag is difficult to directly reproduce in mice. Instead, in the SJ group, we aimed to model the irregular light exposure that varying schedules across the week can create. Subsequent circadian and behavioral phenotyping of these animals identified a number of circadian activity phenotypes and changes in motor function associated particularly with the DLAN condition. While our data suggests that these phenotypes are not due to molecular or cellular changes within the circadian clock, dendritic spine changes in other brain regions raise the possibility that these phenotypes are mediated by changes in neuronal coordination outside of the clock.

Circadian disruption through long-term inappropriate exposure to light is increasingly prevalent in society. Here we have attempted to use mice to model two common means in which inappropriate light exposure occurs in modern societies, namely exposure to dim light at night and irregular exposure to light-dark cycles across the week. While these disruptive lighting regimes are mild in comparison to those used in previous studies (Castanon-Cervantes et al., 2010; McGowan and Coogan, 2013; Deibel et al., 2014), the duration of this period of disruption (approximately 40% of the average life span of a C57BL/6J mouse) was significantly longer than is commonly used. Passive infrared monitoring throughout the duration of the disruption period showed that animals housed in DLAN conditions had weaker activity rhythms and took longer to recover following the cessation of disruptive lighting than control animals or animals under SJ conditions. It is therefore notable that the majority of phenotypes identified in subsequent analyses were found in animals from the DLAN condition, suggesting a correlation between the degree of circadian disruption and the impact upon behavior. This conclusion echoes that of a recent key study from Inokawa et al., in which mice subjected to long-term (600 days) chronic phase advances (which mice were unable to adapt to, leading to severely disrupted rhythmicity) showed increased mortality and a range of immunological defects (Inokawa et al., 2020). Notably, however, chronic phase delays, which the mice were able to resynchronize to, did not cause any subsequent lifespan or immunological phenotypes (Inokawa et al., 2020), implying a relationship between the degree of circadian disruption and the presence of associated phenotypes. Studies of social jetlag in humans have also implicated sleep in such associations. Specifically, while shortened sleep is associated with increased mortality, this association is lost following longer periods of recovery sleep across the weekend (Åkerstedt et al., 2019). Further work is required to ascertain whether such effects are specifically due to disruption of circadian rhythmicity, loss or fragmentation of sleep or a combination of both. Species differences should also be taken into consideration. For example, in humans light exposure promotes waking and arousal, whereas for rodents, light can be both sleep promoting and anxiogenic, depending on the wavelength (Pilorz et al., 2016). The specific disruptive effects of DLAN may therefore vary depending on the species, particularly with regards their effect on sleep. A possible focus of future studies may be to establish which of the phenotypes we report here in mice also persist in other species, e.g., in diurnal animals.

A number of previous studies have demonstrated the impact DLAN has upon behavior. Specifically, exposure to DLAN has been shown to modulate mood and anxiety (Bedrosian et al., 2011, 2013; Aubrecht et al., 2013; Cisse et al., 2016; Chen et al., 2021), circadian function (Shuboni and Yan, 2010) and sleep consolidation (Panagiotou and Deboer, 2020; Panagiotou et al., 2020). Here, we confirm the long-term disruptive influence of DLAN upon circadian activity but also demonstrate an unexpected impact of the condition upon motor function. While grip strength measurements indicate no loss of muscle strength, rotarod tests revealed that animals previously housed under DLAN conditions showed increased motor coordination compared to animals from the other conditions. We also note the possibility of interconnectivity between the circadian and motor coordination phenotypes that we report here. In the present study, we analyzed circadian activity using both traditional wheel-running activity (voluntary motor activity) and passive infrared recordings (total activity). Interestingly we noted a range of significant effects in animals previously housed under DLAN conditions when tested using circadian wheel running which were not found using passive infrared recordings (including increased activity, changes in IV and RA in constant darkness and changes in activity bouts). It is likely that these differences reflect how activity is measured by the two techniques. PIR records all activity within the cage, whereas wheel running only records voluntary motor activity. Wheel running therefore incorporates aspects of motivation and capacity for exercise, and the wheel running-specific phenotypes we demonstrate here may therefore be indicative of changes in associated neuronal or muscular function. It is also possible that the increased aptitude for the rotarod we found in DLAN animals also reflects an increased preference for running wheels, and thus a wider range of wheel running-based circadian phenotypes found in DLAN animals. By contrast it has been noted that running wheels can sometime mask subtle circadian phenotypes (Pritchett et al., 2015) or enhance weaker rhythms (Cuesta et al., 2014). This may be reflected in our dataset in which analysis of PIR data established certain circadian changes not found by wheel running analysis (e.g., shorter period in DLAN animals or reduced RA in SJ animals). Notably, in our disrupted animals we found no significant differences in the molecular oscillations in the SCN (PER2::LUC reporter recordings) or in light input pathways (cFos activation or pupillometry). Regarding the bioluminescence experiment, though, because of the limited statistical power, we cannot rule out subtle changes in SCN clock function. But overall, our data suggest that the circadian phenotypes we report are more likely due to changes in output pathways from the SCN or in extra-clock processes. It has been demonstrated that returning animals to standard LD light cycles following an acute period of disruption will have an ameliorative effect on circadian driven dysfunction (Bedrosian et al., 2013). However, the results we present here suggest that following a prolonged disruption period certain changes will persist outside of the core clock.

As noted above, previous studies have associated DLAN exposure with changes in anxiety and mood (Bedrosian et al., 2011, 2013; Aubrecht et al., 2013; Cisse et al., 2016; Chen et al., 2021). In contrast, we found no evidence of changes in anxiety or cognition in animals from DLAN conditions. We note several possible reasons for this. Firstly, to compensate for purely acute effects, we performed phenotyping following a 2-week period of re-acclimatization to standard LD conditions. This contrasts with previous studies which perform phenotyping immediately after or during the DLAN period (Bedrosian et al., 2011; Cisse et al., 2016). Secondly, the animals used here were subjected to disruptive lighting conditions in group housed conditions, compared to other studies in which animals are often singly housed for the disruption period (Bedrosian et al., 2011; Cisse et al., 2016). Although the evidence for social entrainment in rodents is inconclusive (Refinetti et al., 1992; Paul et al., 2014), it is certainly possible that group housing of animals may modulate their circadian rhythms, behavior, and brain function throughout the DLAN period. Finally, our analysis revealed that the disruptive effect of DLAN was most pronounced during the first 5 months of exposure to the condition. Following this initial period, the disruptive effect of the DLAN condition stabilized, suggesting that the animals underwent a period of acclimatization to the condition. Previous studies which suggested that periods of DLAN can cause behavioral changes, often subjected animals to periods of disruption shorter than 5 months (Bedrosian et al., 2011, 2013; Aubrecht et al., 2013; Cisse et al., 2016; Chen et al., 2021). It is therefore possible that the unexpected lack of cognitive or anxiety phenotypes we found in our disrupted animals was due to their acclimatization to the disruptive conditions, thus mitigating the detrimental impacts of the disruption. Sleep has been suggested to undergo compensatory mechanisms during prolonged exposure to DLAN (Panagiotou et al., 2020) and the analysis we show here suggests that a similar mechanism could exist for circadian activity.

In addition to changes in circadian rhythmicity and motor function, we found an elevation in cortical and hippocampal dendritic spines in mice from DLAN conditions. In contrast, previous studies in hamsters have observed a decrease of hippocampal dendritic spines following exposure to DLAN (Bedrosian et al., 2011; Aubrecht et al., 2013). It is notable, however, that this DLAN-induced spine decrease was reversible, with hippocampal spine numbers recovering to normal levels following 2 weeks of exposure to standard LD conditions (Bedrosian et al., 2013). A detailed analysis of the increase in spine numbers in our mice demonstrated that, in the hippocampus, the elevation in spine numbers was due to an increase in immature/developing spines (Risher et al., 2014). It is therefore possible that this increase in immature spines reflects an overcompensation to the DLAN condition in our animals. While aging and behavioral changes are commonly associated with decreased dendritic spine numbers (Norris et al., 1998; Banks et al., 2018), increased spine numbers have also been correlated with changes in behavior (Banks et al., 2020). It is therefore probable that the elevated spine numbers we observed are linked to the motor and circadian changes we found in our DLAN-exposed mice. Further studies are needed to establish which networks and brain regions underlie these changes.

The study that we present here is one of very few which have analyzed the effect of circadian disruption over a long period of time. Short term studies have often used severe conditions such as rotating jetlag or non-entrainable conditions and have demonstrated the detrimental impact of these conditions upon both physiology and behavior (Castanon-Cervantes et al., 2010; Loh et al., 2010; Karatsoreos et al., 2011; Deibel et al., 2014; West et al., 2017). Notably however the impact of such conditions is more severe in aged animals compared to younger animals (Davidson et al., 2006). The effect of long-term periods of circadian disruption (>6 months) have less commonly been investigated, and those studies which have been performed have used non-entrainable conditions to show the impact of such conditions upon fitness and lifespan (Spoelstra et al., 2016; Inokawa et al., 2020). However, these severe conditions are not representative of the milder long-term disruptive conditions experienced in the modern world, such as DLAN. The study we present here is, to our knowledge, the first to examine the chronic application of this mild circadian disruption and its impact upon behavior. Our findings contrast with those of previous studies that have shown that severe disruption can increase mortality and adversely affect physiology. However, taken together, our data highlights the long-term impact that chronic, mild circadian disruption can have, particularly in relation to exposure to DLAN. Although our data indicates that animals can acclimatize to long-term exposure to DLAN, changes in motor function, circadian rhythmicity and dendritic spines are still observed following both this acclimatization and a subsequent recovery period in standard lighting conditions. While previous studies have demonstrated the health consequences of acute DLAN, the data we have presented here highlight hitherto unknown long-term impacts of such mistimed exposure and how they may impact the process of healthy aging. Given the current prevalence of illumination at night in the modern world, our results call for further work to establish whether similar effects can occur in humans and impact healthy aging.

## Data Availability Statement

The original contributions presented in the study are included in the article/Supplementary Material, further inquiries can be directed to the corresponding authors.

## Ethics Statement

The animal study was reviewed and approved by Animal Care Committees of McGill University and Douglas Mental Health University Institute, in accordance with guidelines of the Canadian Council on Animal Care (for work done in Montreal), and according to United Kingdom Animals (Scientific Procedures) Act 1986 under PPL PE4ED9D2C in accordance with the University Policy on the Use of Animals in Scientific Research (for work done in Oxford).

## Author Contributions

SP, SB, RF, NC, and GB contributed to conception and design of the study. TD, SS, AF, M-ÈC, MS, CP, CM, and GB performed the experiments. TD, SS, AF, M-ÈC, and GB performed the analyses. TD, SS, NC, and GB wrote the manuscript. All authors contributed to manuscript revision, read, and approved the submitted version.

## Conflict of Interest

The authors declare that the research was conducted in the absence of any commercial or financial relationships that could be construed as a potential conflict of interest.

## Publisher’s Note

All claims expressed in this article are solely those of the authors and do not necessarily represent those of their affiliated organizations, or those of the publisher, the editors and the reviewers. Any product that may be evaluated in this article, or claim that may be made by its manufacturer, is not guaranteed or endorsed by the publisher.

## Footnotes

1
http://rsbweb.nih.gov/ij/
