# Supplementary material for: Chronic Exposure to Dim Light at Night or Irregular Lighting Conditions Impact Circadian Behavior, Motor Coordination, and Neuronal Morphology
Source: Front Neurosci. 2022 Apr 13;16:855154. doi: 10.3389/fnins.2022.855154 (PMC9043330; doi:10.3389/fnins.2022.855154)
Supplement: Supplementary file 1 [file Data_Sheet_1.pdf]

Supplementary Material

Supplementary Table 1. Summary of wheel running parameters under 12:12LD, DD and LL for all three chronic lighting treatment groups.

	12:12LD			DD			LL		
	LD (n=8)	DLAN (n=12)	SJ (n=11)	LD (n=8)	DLAN (n=12)	SJ (n=9)	LD (n=7)	DLAN (n=12)	SJ (n=10)
Period (h)	24.0 ± 0.00	24.0 ± 0.00	24.0 ± 0.00	23.88 ± 0.06	23.86 ± 0.03	23.89 ± 0.06	24.95 ± 0.47	25.25 ± 0.11	25.13 ± 0.22
Alpha (h)	11.63 ± 0.25	11.58 ± 0.17	11.6 ± 0.14	11.76 ± 0.30	11.63 ± 0.48	12.33 ± 0.41	6.12 ± 1.42	6.26 ± 0.44	6.58 ± 0.83
(Subjective) day activity (counts)	***458.6 ± 147.1***	***1050 ± 173.5***	***445.3 ± 72.62***	***1142 ± 205.4***	***2423 ± 360.7***	***1380 ± 194.9***	344.4 ± 142.3	480.3 ± 81.60	746.8 ± 355.8
(Subjective) night activity (counts)	7937 ± 2642	16534 ± 2230	12100 ± 2690	***8251 ± 3134***	***18190 ± 3649***	***19123 ± 2400***	***1425 ± 410.4***	***3851 ± 453.9***	***2289 ± 703.9***
Total activity (counts)	8395 ± 2734	17584 ± 2241	12545 ± 2693	***9393 ± 3305***	***20614 ± 3729***	***10502 ± 2393***	***1770 ± 531.9***	***4332 ± 502.1***	***3036 ± 961.3***
(Subjective) day activity (%)	6.96 ± 1.54	7.06 ± 1.34	5.75 ± 1.63	18.01 ± 2.58	14.18 ± 2.13	18.67 ± 3.28	16.47 ± 4.25	11.24 ± 1.76	21.78 ± 4.35
Intradaily variability	***1.24 ± 0.10***	***0.85 ± 0.08***	***0.99 ± 0.11***	***1.32 ± 0.15***	***0.90 ± 0.11***	***1.39 ± 0.12***	1.57 ± 0.11	1.23 ± 0.10	1.63 ± 0.16

Relative amplitude	0.99 ± 0.00	0.99 ± 0.00	0.99 ± 0.01	***0.97 ± 0.01***	***0.99 ± 0.00***	***0.95 ± 0.02***	***0.90 ± 0.06***	***0.96 ± 0.03***	***0.87 ± 0.03***
Interdaily stability	0.70 ± 0.03	0.74 ± 0.03	0.71 ± 0.05	N/A	N/A	N/A	N/A	N/A	N/A
Number of bouts	52.75 ± 3.77	42.00 ± 4.073	51.27 ± 4.478	54.13 ± 5.09	40.33 ± 4.94	57.11 ± 5.65	28.43 ± 1.875	31.33 ± 1.33	34.10 ± 1.97
Average bout length (minutes)	***49.96 ± 12.06***	***111.9 ± 14.62***	***75.57 ± 14.97***	***53.79 ± 17.00***	***112.7 ± 20.00***	***54.19 ± 12.55***	***73.78 ± 5.79***	***96.31 ± 5.79***	***70.47 ± 6.17***
Average counts per bout	***1564 ± 636***	***4673 ± 796***	***3070 ± 984***	***2216 ± 1046***	***5637 ± 1271***	***2055 ± 647***	***582 ± 215***	***1307 ± 174***	***781 ± 244***
Average peak rate	***57.10 ± 7.85***	***79.98 ± 3.05***	***67.53 ± 4.52***	62.70 ± 7.70	89.27 ± 9.16	67.35 ± 5.68	44.47 ± 6.52	57.05 ± 3.49	48.76 ± 5.70

Notes: After 1 year of ageing in either: 12 h of light, 12 h of dark (LD), dim light at night (DLAN) or a social jetlag protocol (SJ), wheel running patterns were analyzed in all mice under three different lighting conditions: 12 h of light, 12 h of dark (12:12LD), constant dark (DD) and constant light (LL). Values represent mean ± SEM. N/A denotes parameters that cannot be assessed in constant conditions (DD and LL). Values that are bold/italic represent a significant group difference for a given lighting condition (12:12LD, DD, or LL) on wheel running parameters.

Supplementary Table 2. Summary of passive infrared monitoring parameters under 12:12LD and DD for all three chronic lighting treatment groups.

	12:12LD			DD		
	LD (n=6)	DLAN (n=12)	SJ (n=10)	LD (n=10)	DLAN (n=12)	SJ (n=10)
Period (h)	24.0 ± 0.00	24.0 ± 0.00	24.0 ± 0.00	***23.87 ± 0.02***	***23.78 ± 0.02***	***23.86 ± 0.02***
Alpha (h)	12.04 ± 0.06	11.87 ± 0.14	11.89 ± 0.16	12.7 ± 0.19	12.75 ± 0.17	12.44 ± 0.11
(Subjective) day activity (counts)	2389 ± 274	2640 ± 143	2782 ± 234	***2629 ± 201***	***3029 ± 267***	***3609 ± 256***
(Subjective) night activity (counts)	9786 ± 642	9987 ± 567	9777 ± 672	10032 ± 590	11047 ± 688	10770 ± 790
Total activity (counts)	12175 ± 898	12627 ± 628	12559 ± 888	12662 ± 682	14077 ± 841	14380 ± 1021
(Subjective) day activity (%)	19.37 ± 0.86	21.25 ± 1.21	22.06 ± 0.63	***20.84 ± 1.22***	***21.6 ± 1.37***	***25.2 ± 0.69***
Intradaily variability	***1.31 ± 0.06***	***1.21 ± 0.03***	***1.41 ± 0.06***	1.35 ± 0.06	1.25 ± 0.04	1.39 ± 0.04
Relative amplitude	0.72 ± 0.02	0.69 ± 0.02	0.66 ± 0.01	***0.73 ± 0.02***	***0.71 ± 0.03***	***0.64 ± 0.02***

Interdaily stability	0.57 ± 0.02	0.58 ± 0.02	0.51 ± 0.02	N/A	N/A	N/A
Number of bouts	45.64 ± 0.87	46.93 ± 1.55	47.01 ± 1.35	47.13 ± 1.47	48.47 ± 1.38	51.89 ± 1.78
Average bout length (minutes)	14.35 ± 0.6	14.11 ± 0.44	14.62 ± 0.59	14.38 ± 0.39	14.27 ± 0.51	14.73 ± 0.78
Average counts per bout	264.62 ± 17.9	271.33 ± 17.4	268.175 ± 23.8	270.6 ± 16.14	292.5 ± 17.2	280.8 ± 24.7
Average peak rate	24.41 ± 1.23	25.17 ± 1.04	25.57 ± 0.67	24.46 ± 0.75	24.35 ± 0.51	25.09 ± 0.77

Notes: After 1 year of ageing in either: 12 h of light, 12 h of dark (LD), dim light at night (DLAN) or a social jetlag protocol (SJ), passive infrared activity patterns were analyzed in all mice under two different lighting conditions: 12 h of light, 12 h of dark (12:12LD) and constant dark (DD). Values represent mean ± SEM. N/A denotes parameters that cannot be assessed in constant conditions (DD). Values that are bold/italic represent a significant group difference for a given lighting condition (12:12LD or DD) on passive infrared activity parameters.

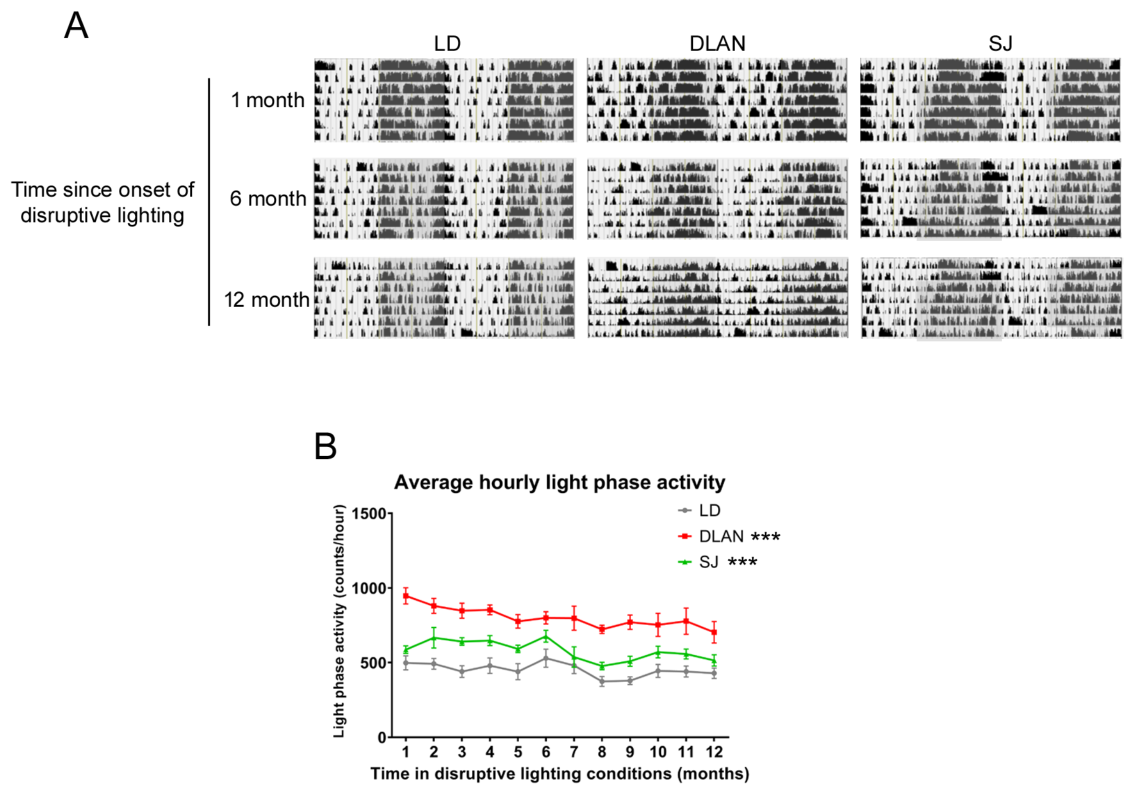

Supplementary Fig. 1. Activity of group housed animals during disruption period. (A) Representative double-plotted actograms of group housed animals, at 1 month, 6 months and 12 months following the onset of disruptive lighting. Actograms represent 7 days of recording. Periods of darkness or dim light are represented in grey. (B) Average counts per hour in the light phase for group housed cages in different lighting conditions.

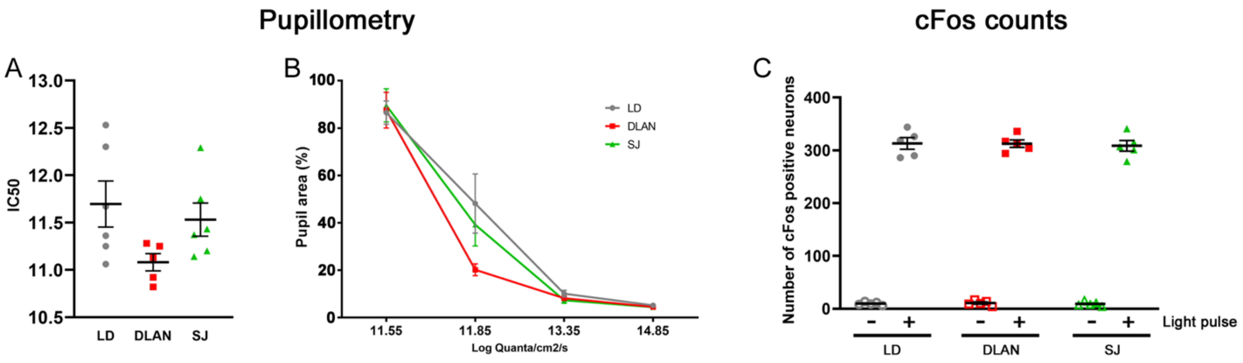

Supplementary Fig. 2. Analysis of light input pathways, following the end of the 12-month lighting disruption period. (**A-B**) Pupillary light reflex measured by dark-adapted pupillometry: rate of constriction (**A**) and pupil constriction across different light intensities (**B**) for mice from disruptive conditions (see Fig. 1 for a description of the three groups). (**C**) Light-induced cFos expression in the suprachiasmatic nucleus (cell counts of immuno-labelled cells). One-way ANOVA (with Welch correction, or Kruskal-Wallis test when appropriate), groups: LD, DLAN, SJ. Post-hoc tests conducted with Benjamini-Hochberg corrections when appropriate. All n.s.

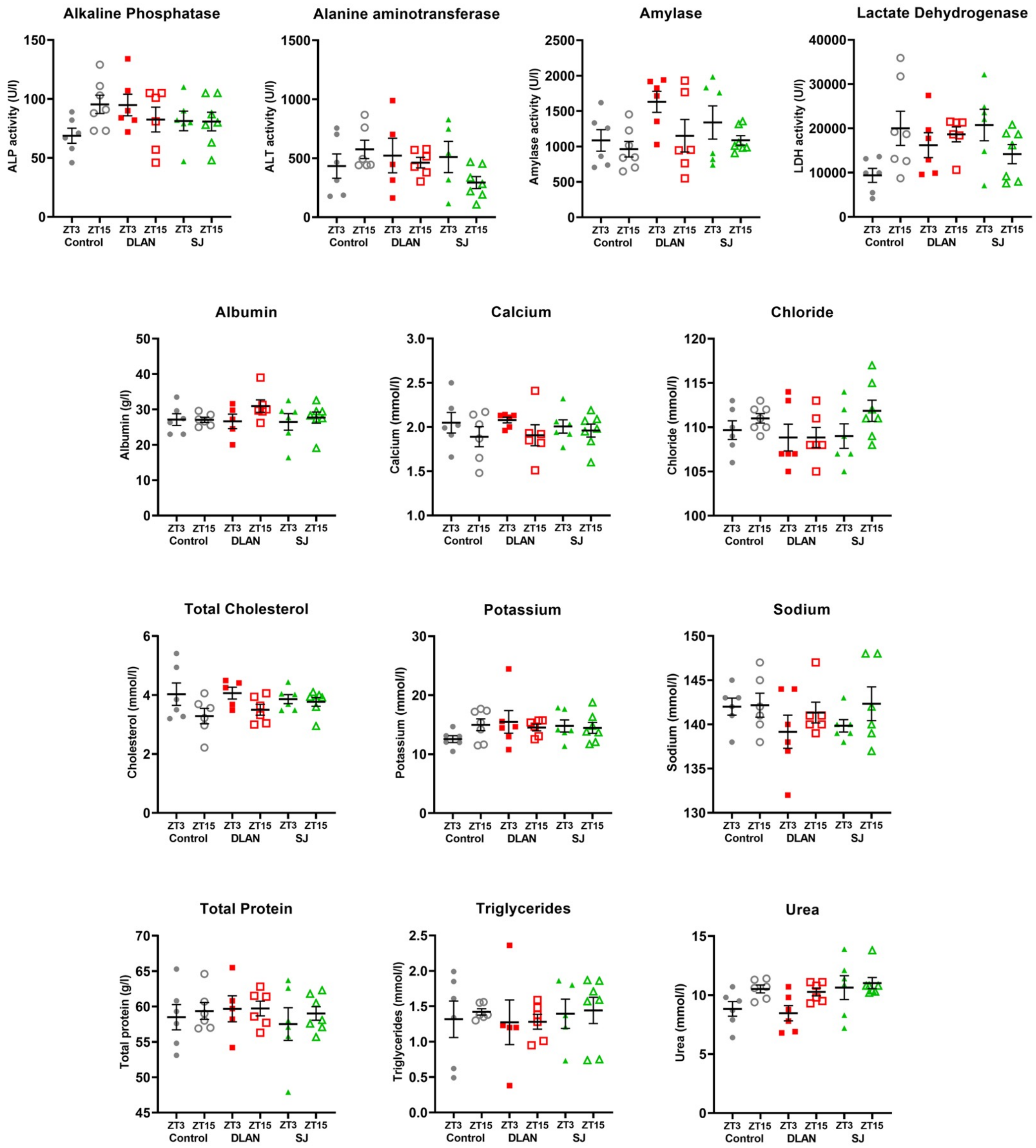

Supplementary Fig. 3. Clinical chemistry measures in the serum of mice from disruptive conditions, following the end of the 12-month lighting disruption period, at Zeitgebeer (ZT) 3 and 15. Measures include activity levels of alkaline phosphatase, alanine aminotransferase, amylase or lactate dehydrogenase, and levels of albumin, calcium, chloride, cholesterol, potassium, sodium, protein, triglycerides or urea. One-way ANOVA (with Welch correction, or Kruskal-Wallis test when appropriate), groups: LD, DLAN, SJ. Post-hoc tests conducted with Benjamini-Hochberg corrections when appropriate. All n.s.
